# Unwanted Consensual Sex Among College Students: What Makes an Individual More Likely to Consent to Sex They Do Not Want?

**DOI:** 10.3390/bs15070981

**Published:** 2025-07-19

**Authors:** Kathryn J. Barnhart, Katelin E. Leahy, Mikhila N. Wildey

**Affiliations:** 1 School of Interdisciplinary Health, Grand Valley State University, Allendale, MI 49401, USA; 2 Independent Researcher, East Lansing, MI 48824, USA; leahykatelin@gmail.com; 3 Department of Psychology, Grand Valley State University, Allendale, MI 49401, USA; wildeymi@gvsu.edu

**Keywords:** unwanted consensual sex, gender, communication

## Abstract

Sexual consent is essential, but in some instances an individual may consent to sexual behaviors that are unwanted. The likelihood of unwanted consensual sex may be influenced by multiple social and individual factors. The current study examined engagement in unwanted consensual sex based on demographics, relationship and/or hookup history, and sexual communication characteristics among college students. This cross-sectional study was conducted utilizing a random sample of college students at one midwestern university. Data was collected via an online survey, including questions about sexual behavior, communication, and sexual consent. Basic frequencies and bivariate and multivariate analyses were conducted. This random sample included 1263 undergraduate students. Level of comfort communicating about sex was negatively associated with unwanted consensual sex. Gender was significantly associated with the likelihood of unwanted consensual sex and was a significant predictor of unwanted consensual sex among this sample. Unwanted consensual sex may vary based on individual characteristics, like gender. The current findings may help guide future studies on unwanted consensual sex, as well as continued sex education programming on college campuses to increase comfort when communicating about sexual behaviors.

## 1. Introduction

Sexual experiences are often described as either being consensual or nonconsensual, but this dichotomy conflates consenting with wanting to have sex and nonconsenting with not wanting to have sex (Muehlenhard & Peterson, 2005; Peterson & Muehlenhard, 2007a). Definitions of sex among college samples may be situational, such that they are influenced by personal motives, and confidence in the definition may differ based on experience and perceptions of personal behaviors versus the behaviors of others (Peterson & Muehlenhard, 2007b; Sewell & Strassberg, 2015). Within consensual sexual experiences, desire is an important factor. Thus, unwanted consensual sex is often defined as consensual sex where desire is absent in at least one partner and there is no immediate pressure to consent to sexual activity (Katz & Tirone, 2010; O’Sullivan & Allgeier, 1998; Peterson, 2024). For example, some individuals consent to unwanted sex for their partner’s pleasure, and others may consent because they want to increase or maintain intimacy with a partner (Katz & Schneider, 2015; O’Sullivan & Allgeier, 1998). However, if unwanted sex is thought of on a continuum, then not all aspects of this example are unwanted, as the individual wanted their partner’s pleasure and to maintain intimacy (Peterson, 2024). Other instances of nonconsensual sex may be due to pressure from a partner. In these instances, consent is given as a result of coercion, relationship conflict, or violence (Impett & Peplau, 2002; Jozkowski & Peterson, 2013).

Examining consent conversations between partners is often contextualized by sexual scripts or the culturally guided progression of heteronormative sexual interactions (Gagnon & Simon, 2009). Sexual script theory proposes that sexual experiences and behaviors are socially constructed by scripts and influenced by various social situations (Simon & Gagnon, 1986). Typically, these scripts depict men as sexual initiators (i.e., they express a desire to have sex), and women are seen as sexual gatekeepers (i.e., they decide if sex will occur) (Jozkowski & Peterson, 2013). It is important to understand how individuals discuss sexual behaviors beyond consent, considering the differing expectations that are levied upon men and women (e.g., Jozkowski & Humphreys, 2014). Given the extant literature on consent and sexual scripts, it might be the case that women more often than men consent to unwanted sex, particularly when a conversation about acceptable sexual behavior is difficult. The majority of research on unwanted consensual sex focuses on sexual script theory, which ties to a social constructionist view of gender, mostly looking at heterosexual scripts (Peterson, 2024).

When considering demographic variables, previous research estimates that approximately a third of Black women and White women admitted to engaging in unwanted consensual sex, which is similar to findings among samples of predominantly white women (Blythe et al., 2006). Limited research to date has examined age, race/ethnicity, and sexual orientation specifically in relation to frequency of unwanted consensual sex in college (e.g., Goodman et al., 2019). There is therefore a need for additional investigation of possible associations between demographic variables and the likelihood of unwanted consensual sex among larger samples.

The majority of the existing work on unwanted consensual sex contain samples of college students (Bay-Cheng & Bruns, 2016; Katz & Schneider, 2015; Katz & Tirone, 2009, 2010; Katz et al., 2012; Muehlenhard et al., 2016; O’Sullivan & Allgeier, 1998). However, few studies have explored unwanted consensual sex specifically during casual sexual encounters (Katz & Schneider, 2015; Peterson, 2024). Hookups are frequently defined as casual encounters and as common among college students (Garcia et al., 2012). Hookup behaviors may be facilitated by the university student environment, which often provides close living quarters, access to a variety of potential partners, and a party atmosphere (Bogle, 2008; Pham, 2017; Wade, 2017). Previous studies have shown that hookups occur following alcohol consumption, which is also common behavior among university students, with those who binge drink having an increased likelihood of reporting a hookup (e.g., Garcia & Reiber, 2008; Garcia et al., 2012; Wade, 2017). Although young adults report engaging in consensual sexual behavior while intoxicated, the association between the level of alcohol consumed and ability to consent is under-researched (Herbenick et al., 2019; Jozkowski & Wiersma, 2015; Muehlenhard et al., 2016). Unwanted consensual sex during casual sexual events among college students is important to explore further, given that the college environment may allow for more casual and open sexual experiences that some may consider a normative and fun form of sexual exploration (Garcia et al., 2012; Wade, 2017).

Understanding who among college students is having unwanted consensual sex, how often they are having it, and which individuals are more likely to endorse this behavior can provide researchers information about groups that might be at increased risk for outcomes related to unwanted consensual sex and can help to address these outcomes. The current cross-sectional study aims to explore (1) how the frequency of unwanted consensual sex might differ based on an individual’s demographic characteristics, (2) the association between comfort in communicating with relationship and hookup partners and rates of unwanted consensual sex, and (3) possible predictors of unwanted consensual sex.

## 2. Materials and Methods

### 2.1. Procedure and Participants

All study procedures were reviewed by the primary author’s institutional review board prior to implementation. To be included in this study, participants needed to identify as age 18 or older and be currently enrolled in college courses. Recruitment emails were sent to 6500 randomly selected undergraduate students at a large midwestern university through the university’s institutional analysis office. Consent was obtained via Qualtrics prior to accessing the electronic survey.

Of the 6500 students who were invited to participate in the survey, 1263 completed the survey (i.e., a response rate of 19.4%). The current study excluded 165 participants who answered that they had never engaged in any type of sexual activity in their lifetime, given that the focus of the current project is on behaviors related to sexual activity. Participants who identified as man/trans-man or a woman/trans-woman were included in the current subsample. Participants who identified as nonbinary or genderqueer were limited in this sample and therefore excluded (*n* = 14). These exclusions yielded a final sample size of 1084 participants for the current study.

The larger study operationalized sex and hookups based on participants’ definitions. The majority of participants in the larger sample considered penile-vaginal intercourse and penile-anal intercourse as part of their definitions of sex, and more than half also included oral sex (giving or receiving) in their definition. The overall sample considered several behaviors ranging from deep kissing to penile-vaginal sex as potentially part of hooking up. Survey questions specific to this study assessed participant demographics, unwanted consensual sex, and level of comfort communicating about sexual behaviors. Variables of interest included gender, age, race/ethnicity, sexual identity, and level of comfort in communicating about sexual behaviors as potential factors in the likelihood of ever having engaged in unwanted consensual sex. To measure the frequency with which participants endorsed engaging in unwanted consensual sex, participants were asked how often they consent or agree to have sex even though they’d rather not have it, which was rated on a 4-point scale (1 = never, 2 = rarely, 3 = sometimes, 4 = often).

To measure comfort in communicating about sexual behavior, participants were asked about the level of comfort they feel communicating with a partner about what they want or do not want to do sexually. The same question was asked twice, once when thinking about relationships and again when thinking about hookups. Questions based on hooking up or being in a relationship were only addressed to respondents who indicated that they had experience with these situations. Both questions were rated on a 5-point scale (1 = not at all comfortable, to 4 = very comfortable, to 5 = I’ve never been in a relationship before). Each question was examined independently, rather than taking a mean.

### 2.2. Analysis

Participant characteristics were analyzed using basic frequencies. Demographic variables of interest were selected based on consistency with prior work (gender and race) and limited examination (age and sexual orientation) (Blythe et al., 2006; Goodman et al., 2019). To provide enough power for statistical tests, variables of interest were recoded accordingly. Given the small *n* for some race/ethnicity categories (e.g., Black, Hispanic/Latino, Asian or Pacific Islander, Native American, multiracial), for the purposes of these analyses, the variable for race/ethnicity was dichotomized into White and people of color. Due to a low number of participants identifying within each sexual minority group, the sexual identity variable was dichotomized into heterosexual and sexual minority. For each categorical demographic group (i.e., gender, race/ethnicity, and sexual identity) independent-sample *t*-tests were conducted to examine potential differences among groups that engaged in unwanted consensual sex. We also examined potential differences among these groups in relation to comfort in communicating about sexual behaviors with hook-up and relationships partners. Correlations were conducted to determine if there was an association between frequency of unwanted consensual sex and (1) age and (2) levels of comfort regarding communicating about sexual behaviors with hook-up and relationships partners, respectively. Finally, we conducted a multiple regression analysis to examine which variables of interest (gender, age, race/ethnicity, sexual orientation, level of comfort in communicating about sexual behaviors) best predict engagement in unwanted consensual sex controlling for prior hookup experience. The interaction between gender and communication with hookup partners was explored to assess how people communicate with both relationship and hookup partners, and this analysis excluded participants who indicated that they had never been in a relationship (*n* = 45).

Prior to the analyses, only scale options of 1–4 were used for the variable “level of comfort communicating about sexual behavior with relationship partners,” given that a score of 5 indicated that the person had never been in a relationship (see above for scale anchors). In addition, age, level of comfort regarding communicating with relationship partners, and level of comfort regarding communicating with hookup partners were grand mean-centered. Gender, race, sexual orientation, and hookup history were effect-coded (i.e., men = 1, women = −1; White = 1, people of color = −1; heterosexual = 1, sexual minority = −1; have hooked up before = 1, never hooked up = −1).

## 3. Results

As seen in Table 1, which presents the demographic details of the current sample, the majority of the current sample consisted of women (69.8%, *n* = 757) who were ages 18–22 (85.3%, *n* = 925), were White (88.5%, *n* = 959), and identified as heterosexual/straight (88.2%, *n* = 956). Forty-five participants indicated they had not been in a relationship, which is worth noting because participants could have had many kinds of experiences (i.e., had hookup and relationship partners or only been in relationships). Finally, 69.3% (*n =* 751) reported engaging in hookup behaviors, as defined by the participant.

Regarding additional variables of interest in the current study, Table 2 lists frequency data for unwanted consensual sex and the two comfort with communication items. As seen in the table, approximately half of the participants (49.3%) who responded said they have never consented to sex when they did not want to have it. For those who reported having ever engaged in unwanted consensual sex, 28% reported that this was rare, 14.7% reported that it happens sometimes, and 8% said that consenting to unwanted sex happens often. When asked about their levels of comfort communicating about sexual behaviors to a relationship partner, 72.3% felt very comfortable, and 21.4% felt somewhat comfortable (*M* = 3.65, *SD* = 0.63). Only 67.3% report being very or somewhat comfortable communicating about sexual behaviors with hookup partners, with 32.7% reporting little or no comfort (*M* = 2.86, *SD* = 1.00).

A series of independent sample *t*-tests were conducted to compare the likelihood of unwanted consensual sex among demographic groups (i.e., gender, age, race/ethnicity, and sexual orientation). Cohen’s *d* effect sizes were calculated to illustrate the magnitude of difference between each group, and these effect sizes are reported in Table 2. Standard conventions for the interpretation of Cohen’s *d* effect sizes were used, such that a small effect was defined as *d* = 0.20, a medium effect as *d* = 0.50, and a large effect as *d* = 0.80. As seen in Table 2, a significant difference was found between gender and frequency of unwanted consensual sex: *t*(1068) = −4.18, *p* < 0.001), with women consenting to unwanted sex more often than men (Cohen’s *d* effect size = 0.28). There were no significant differences between racial/ethnic group and unwanted consensual sex: *t*(1066) = −1.57, *p =* 0.12, or sexual identity and unwanted consensual sex: *t*(1067) = −1.71, *p =* 0.09. There was no significant association between age and unwanted consensual sex: *r =* 0.04, *p* = 0.15, *n* = 1062.

Regarding the items examining comfort in communication, as seen in Table 2, there were no significant group differences for nearly all items. The exception was for the item examining comfort in communication during hookups, where people of color were significantly more likely to report comfort in communicating during hookups than White participants: *t*(167.7) = −4.58, *p* < 0.001 (*d* = −0.41).

For participants who reported ever engaging in hookup behaviors, there was a negative association between level of comfort in communicating about sexual behaviors with a relationship partner and the likelihood of unwanted consensual sex: *r* = −0.08, *p* = 0.03, *n* = 714. Similarly, there was a negative association between level of comfort in communicating about sexual behaviors with a hookup partner and likelihood of engaging in unwanted consensual sex: *r* = −0.12, *p* = 0.001, *n* = 713. These results indicate that greater likelihood of engaging in unwanted consensual sex is associated with less comfort communicating with a relationship or hookup partner about sexual behaviors.

Among participants who reported never being in a relationship, there was a non-significant association between sexual communication comfort and how often they engage in unwanted consensual sex: *r* = 0.24, *p* = 0.18, *n* = 33. Among participants reporting never engaging in hookup behaviors, there was no significant association between level of comfort communicating about sexual behaviors and likelihood of engaging in unwanted consensual sex: *r* = −0.02, *p* = 0.67, *n* = 312.

Table 3 presents the results from the regression model. In this model, controlling for those who have never hooked up, women were more likely than men to have consented to unwanted sex: (*β* = −0.13). All other variables were not significant, including the interaction.

## 4. Discussion

Overall, the current study found an approximately even split between participants who have and participants who have not engaged in unwanted consensual sexual behaviors. Similar to previous studies, women reported unwanted consensual sex more often than men (Impett & Peplau, 2002; Katz et al., 2012; O’Sullivan & Allgeier, 1998). These findings are consistent with the existing literature, suggesting that gendered sexual scripts may have an influence on an individual’s behavior, particularly with women acting as the gatekeepers for sexual encounters or disregarding their own desires for those of their partners (Katz & Tirone, 2009; Katz & Schneider, 2015; O’Sullivan & Allgeier, 1998; Scappini & Fioravanti, 2022). These gendered sexual scripts may also be examples of what Gagnon and Simon refer to as interpersonal scripts, which facilitate the occurrence of a sexual encounter taking place, in this case, an encounter that was unwanted by the woman even when consensual (1986).

Participants in the current sample reported lower levels of comfort communicating with hookup partners and higher levels of comfort communicating with their relationship partners about sexual behaviors. Among those who had previous experience with both relationship and hookup partners, the less comfortable they were in communicating about sexual behavior, the more likely they were to engage in unwanted consensual sex. This finding suggests that when people are uncomfortable talking about what they were willing or not willing to do sexually with their partner, they may engage in unwanted consensual sex more often. Prior research on communication of consent shows that women more often use verbal strategies to express consent, whereas men use more nonverbal strategies to both express and interpret consent (Jozkowski & Peterson, 2013). The degree of comfort with communication about the specific sexual behaviors an individual is willing to engage in may be tied to their experiences with both hookup and relationship partners. However, additional research is needed to understand how types of sexual partners influence sexual communication strategies and what this means for likelihood of engaging in unwanted consensual sex.

Finally, the current study found that among those who engage in hookup behaviors and across the various demographic and communication predictors, women were more likely to engage in unwanted consensual sex than men. Consistent with the findings of Goodman et al. (2019), age, sexual identity, and race were not significant predictors of consensual unwanted sex. While unwanted consensual sexual encounters are not always categorized as riskier sexual behaviors, regular occurrences of unwanted but consensual sexual behaviors may increase the likelihood of negative outcomes, such as poor psychological, sexual, or relationship factors for individuals (Peterson, 2024).

## 5. Limitations/Future Research

There are limitations in the current study. The sample was relatively homogenous and relied on self-report. Participants who identify as nonbinary or gender-queer were excluded from the current analysis due to low sample size (*n* = 14). The homogeneous sample and the exclusion of nonbinary and gender-queer participants impacts generalizability to all members of the campus community and presents a gap in our current findings. Additional research is needed to specifically explore unwanted consensual sex among more diverse samples. Recall bias may be present due to the variations in time between sexual events and the survey. In addition, it is worth noting that specific reasons for engaging unwanted consensual sex were not provided by participants. Reasons for engaging in unwanted consensual sex among men and women have been investigated in previous research and may also be applicable to various participants in the current sample (Khera et al., 2022; Peterson & Muehlenhard, 2007a).

Future research is needed to further disentangle the motivations and situational contexts that might drive this behavior, as well as determine possible negative outcomes. Longitudinal mixed-methods studies, possibly including event-based diary research, to further explore trends and consistency of engaging in unwanted consensual sex are needed to explain the intricacies of this type of sexual encounter and identify characteristics that might make unwanted consensual sex a norm. Understanding the factors associated with the likelihood of unwanted consensual sex can help researchers, therapists, counselors, and educators identify strategies for discussing this behavior for adaptive reasons (relationship maintenance) versus strategies for discussing with those at risk for some of the potentially negative outcomes (relationship violence). Our findings align with previous recommendations for practical application focused on couples and communication to encourage conversation about reasons for wanting or not wanting sex and sexual consent (Peterson, 2024). Beyond couples, individual comfort levels may be lower when communicating with hookup partners. The current findings reinforce the need for continued sex education programming on college campuses to increase the likelihood of comfort when communicating about sexual behaviors, especially with casual or hookup partners. Gender-based programming to address communication discomfort and increase self-advocacy strategies among women may help to reduce the frequency of unwanted consensual sex among this group. Sex education programming focused on consent, communication, and self-advocacy may be delivered through freshman/new student orientations, university wellness centers, applicable classrooms, and other university level policies and resources that support a wholistic approach to students’ health and wellbeing.

## Figures and Tables

**Table 1:** Demographic characteristics of participants (*N* = 1084).

Characteristic	Category	*n* (%)
Gender Identity	Man	327 (30.2)
	Woman	757 (69.8)
Age	Overall Mean (*SD*)	20.9 (3.4)
	18–22	925 (85.3)
	23+	137 (12.7)
Race	White or Caucasian (Non-Hispanic)	959 (88.5)
	Hispanic or Latino	33 (3.0)
	Black or African American	35 (3.2)
	Asian or Asian American	17 (1.6)
	Native American/Alaska Native	4 (0.4)
	Multiracial	28 (2.6)
	Other	6 (0.6)
Sexuality	Heterosexual/Straight	965 (88.2)
	Gay	18 (1.7)
	Lesbian	16 (1.5)
	Bisexual	66 (6.1)
	Queer	10 (0.9)
	Asexual	3 (0.3)
	Other	14 (1.3)
Relationship History	Been in a Relationship	1039 (95.8)
	Never in a Relationship	45 (4.2)
Hookup History	Never Engaged in “hooking up”	333 (30.7)
	Engaged in “hooking up”	751 (69.3)

**Table 2:** Frequency and trend data for unwanted consensual sex and comfort with communication items.

	*n* (%)	Gender (Men > Women)	Race (White > POC)	Sexual Identity (Straight > Sexual Minority)
Frequency of unwanted consensual sex				
Never	527 (49.3%)			
Rarely	300 (28.0%)			
Sometimes	157 (14.7%)			
Often	86 (8.0%)			
Cohen’s *d* effect size		−0.28 *	−0.15	−0.16
Comfort communicating with hookup partners				
Not at all comfortable	134 (12.5%)			
A little comfortable	216 (20.2%)			
Somewhat comfortable	387 (36.1%)			
Very comfortable	334 (31.2%)			
Cohen’s *d* effect size		0.12	−0.41 *	−0.03
Comfort communicating with relationship partners				
Not at all comfortable	12 (1.2%)			
A little comfortable	54 (5.2%)			
Somewhat comfortable	222 (21.4%)			
Very comfortable	751 (72.4%)			
Cohen’s *d* effect size		−0.01	0.04	0.03

*Note*: * *p* < 0.001 in an independent-sample *t*-test that compared group differences. POC = people of color.

**Table 3:** Regression model predicting frequency of unwanted consensual sex as a function of demographics, communication, and the interaction between gender and communication.

	*b*	*se*	*β*	*t*	Δ*R*^2^
Intercept	1.807	0.063		28.796 *	
Have hooked up	0.18	0.034	0.17	5.28 *	0.024 *
Gender	0.128	0.033	0.12	−3.91 *	
Age	0.011	0.009	0.04	1.28	
Sexual Orientation	0.062	0.047	0.04	−1.31	
Race/Ethnicity	0.078	0.047	0.05	−1.66	
Comf. comm. with hookup partners	0.068	0.037	0.07	−1.88	
Comf. comm. with relationship partners	0.039	0.051	0.03	−0.78	0.032 *
Gender*comf. comm. with hookup partners	0.062	0.034	0.06	1.84	0.003

*Note*: * *p* < 0.001, Comf. = comfort; Comm. = communicating.

## Data Availability

Due to ethical reasons, raw data will be made available by the authors upon request.
